# State of *Drosophila melanogaster* Ovaries after a Full Cycle of Gametogenesis under Microgravity Modeling: Cellular Respiration and the Content of Cytoskeletal Proteins

**DOI:** 10.3390/ijms22179234

**Published:** 2021-08-26

**Authors:** Maria A. Usik, Maria A. Golubkova, Irina V. Ogneva

**Affiliations:** 1 Cell Biophysics Laboratory, State Scientific Center of the Russian Federation Institute of Biomedical Problems of the Russian Academy of Sciences, 76a, Khoroshevskoyoe Shosse, 123007 Moscow, Russia; ma_golubkova@mail.ru; 2 Medical and Biological Physics Department, I. M. Sechenov First Moscow State Medical University, 119991 Moscow, Russia

**Keywords:** oogenesis, ovary, simulated microgravity, cell respiration, cytoskeleton, fruit fly

## Abstract

The effect of weightlessness on gametogenesis and the functional state of female germ cells are still poorly understood. We studied the ovaries of *Drosophila melanogaster*, the full development cycle of which (from zygote to sexually mature adults) passed under simulated microgravity by a random positioning machine. The rate of cellular respiration was studied by polarography as a parameter reflecting the functional state of mitochondria. The content of cytoskeletal proteins and histones was determined using Western blotting. The relative content of mRNA was determined using qRT-PCR. The results obtained indicated an increase in the rate of cellular respiration under simulated microgravity conditions during the full cycle of gametogenesis in *Drosophila melanogaster* due to complex I of the respiratory chain. In addition, an increase in the contents of actin cytoskeleton components was observed against the background of an increase in the mRNA content of the cytoskeleton’s encoding genes. Moreover, we observed an increase in the relative content of histone H3 acetylated at Lys9 and Lys27, which may explain the increase in the expression of cytoskeletal genes. In conclusion, the formation of an adaptive pattern of functioning of the *Drosophila melanogaster* ovaries that developed under simulated microgravity includes structural and functional changes and epigenetic regulation.

## 1. Introduction

From the perspective of increasing the duration of space flights, questions have arisen concerning the need to maintain reproductive health. Nevertheless, there are gaps in understanding the effect of real microgravity on the function of the reproductive system. There are a number of difficulties in studying the reproduction process during space flight, in connection with which research with the use of model experiments, including those on animals, is required.

Data for different species are scarce; however, it is known that under space flight conditions, there is a possibility of obtaining offspring from the fruit fly *Drosophila melanogaster*, Medaka fish, and the amphibian *Pleurodeles waltl*. Nevertheless, some developmental anomalies have been noted [1,2,3,4]. At the same time, it was not possible to obtain pregnancy in mammals, since there were difficulties with fertilization and embryo development in the early stages of embryogenesis [5,6].

Studying the full cycle of gametogenesis may be of particular interest from the point of view of maintaining a species. In the implementation of the normal process of maturation and differentiation of germ cells, programmed cell death plays an important role [7,8]. For *Drosophila melanogaster*, it was shown that during oogenesis, cells undergo various stage-specific scenarios of cell death, including apoptosis, primarily through the mitochondrial pathway [9,10]. It was reported that cytosolic actin is involved in the process of mitochondria-dependent apoptosis [11]; it is also known that specific interactions between actin and mitochondria mediate the division of mitochondrial networks and mitochondrial transport, contributing to the cellular distribution of mitochondria [12,13]. In addition, the presence of beta-actin-containing structures within the mitochondria and polymerized actin around the mitochondria is necessary to maintain the membrane potential and optimal transcription of mitochondrial DNA. It was shown that the absence of beta-actin disrupted the activity of OXPHOS and decreased cellular levels of ATP [14]. Moreover, smooth muscle alpha-actin and cytosolic gamma-actin do not functionally compensate for the loss of beta-actin; in isolated mtDNA, only beta-actin was identified, not gamma-actin [15], which may indicate the specific role of beta-actin in maintaining transcription mtDNA, its copy numbers, and mitochondrial membrane potential [14].

There is also a relationship between the movement of mitochondria during maturation and the dynamic network of microtubules. Reorganization of the cytoskeleton of a cell during maturation can affect the clustering of mitochondria, which has been demonstrated in a space experiment. Thus, during the cultivation of human lymphocyte (Jurkat) cells under conditions of space flight and cells from *Drosophila melanogaster* insects (Schneider S-1) under conditions of altered gravity created by rotation of the clinostat, similar changes were observed; namely, both cell lines exhibited mitochondrial abnormalities and clustering, which was regarded to occur as a result of the destruction of microtubules and failure of mitochondrial transport along the microtubules [16]. It was also shown that a change in the functional state of mitochondria, in particular, a decrease in the potential of the mitochondrial membrane, can affect the formation of the division spindle in oocytes, which ultimately led to nondisjunction of chromosomes and, consequently, chaotic mosaicism in preimplantation human embryos [17]. Thus, we discuss the mutual influence of the structure of the cytoskeleton and the functional state of the mitochondria. It is well known that under simulated microgravity, the structure of the cytoskeleton and the content of cytoskeletal proteins change in different types of cells, and these changes usually correlate with changes to the cytoskeleton’s gene expression [18,19,20]. In eukaryotes, transcriptional regulators can be RNA interference [21], DNA methylation [22,23], and various chromatin modifications [24], including covalent posttranslational modifications of histones [25,26], in particular, acetylation.

Therefore, we assessed cellular respiration and the content of several mitochondrial proteins and determined the relative content of cytoskeletal proteins and corresponding mRNAs, as well as acetylated forms of histones in the ovaries of *Drosophila melanogaster* fruit flies after exposure to simulated microgravity conditions during the full gametogenesis cycle.

## 2. Results

### 2.1. Cell Respiration

**Figure 1:**
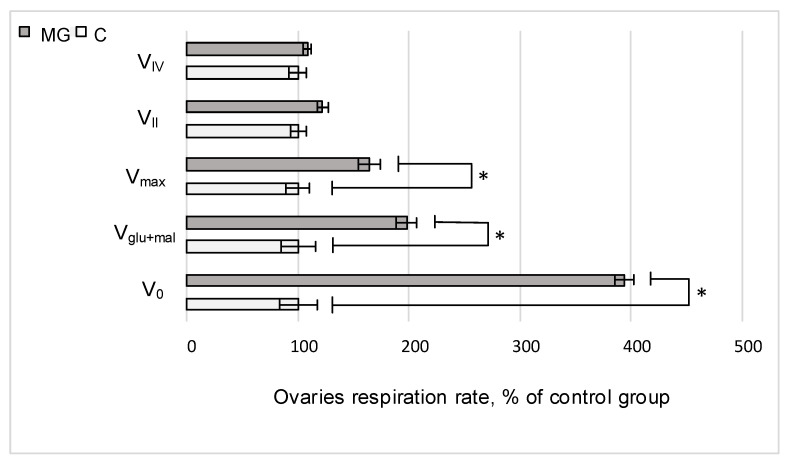
Relative values of the rate of cellular respiration of *Drosophila melanogaster* ovaries. V_0_—cellular respiration rate of permeabilized ovaries; V_Glu+Mal_—the rate of cellular respiration with the addition of 10 mM glutamate + 5 mM malate; V_max_—the maximum rate of cellular respiration after the addition of 2 mM ADP; V_II_—the rate of cellular respiration with the sequential addition of 0.5 mM rotenone (NADH dehydrogenase inhibitor) and 10 mM succinate (succinate dehydrogenase substrate); V_IV_—the rate of cellular respiration upon the sequential addition of 5 mM antimycin (an inhibitor of cytochrome c reductase) and 0.5 mM TMPD + 2 mM ascorbate (artificial substrates of cytochrome c oxidase). C—control group; MG—group of simulated microgravity. * *p* < 0.05 compared to the control group C.

After exposure to simulated microgravity during the full cycle of gametogenesis, the rate of oxygen uptake by permeabilized ovaries, V_0_, increased by 293% (*p* < 0.05); after the addition of substrates of the first complex of the respiratory chain, the respiration rate, V_glu+mal_, was 98% higher (*p* < 0.05) than that of the control group, and the maximum respiratory rate, V_max_, upon the addition of ADP increased by 64% (*p* < 0.05). There were no statistically significant differences between the rates of cellular respiration after inhibition of the first complex of the respiratory chain and the addition of substrates of the second complex, V_II_. In addition, in a similar analysis of the operation of the fourth complex, the rate of cellular respiration in the simulated microgravity group (MG) did not differ from that in the control group C (Figure 1).

### 2.2. Protein Content

**Figure 2:**
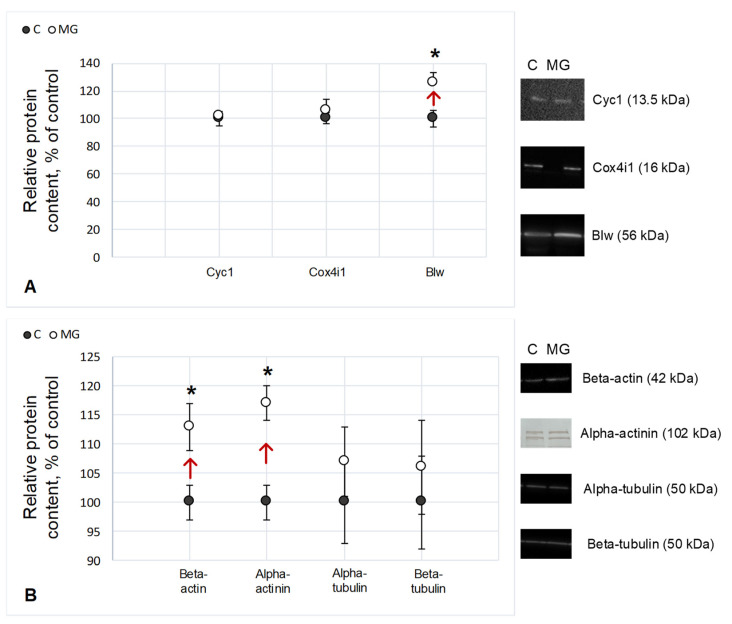
The relative content of proteins with typical Western blots. (**A**) Proteins involved in cellular respiration of *Drosophila melanogaster* ovaries. Cyc1 (13.5 kDa)—cytochrome c-1, a respiratory chain protein located between complexes III and IV; Cox4i1 (16 kDa)—cytochrome c oxidase, a protein of complex IV of the respiratory chain; Blw (56 kDa)—the subunit of ATP synthase F1. (**B**) Cytoskeletal proteins of the *Drosophila melanogaster* ovary. Beta-actin (42 kDa)—microfilament network component; alpha-actinin (102 kDa)—microfilament network component; alpha- and beta-tubulin (50 kDa)—microtubule network components; C—control group; MG—group of simulated microgravity. Arrows indicate the direction of change. * *p* < 0.05 compared to the control group C.

The relative content of cytochrome c and cytochrome c oxidase in the experimental group did not change relative to control levels. At the same time, there was an increase of 26% (*p* < 0.05) in the relative content of the catalytic subunit F1 of ATP synthase (Blw) in the simulated microgravity group (Figure 2A).

The relative content of microfilament proteins beta-actin and actin-binding protein alpha-actinin increased by 13% (*p* < 0.05) and 17% (*p* < 0.05), respectively, relative to control levels when simulating the effects of microgravity. No changes in the protein content of the primary components of microtubules, alpha- and beta-tubulin, were observed (Figure 2B).

### 2.3. mRNA Relative Content

**Figure 3:**
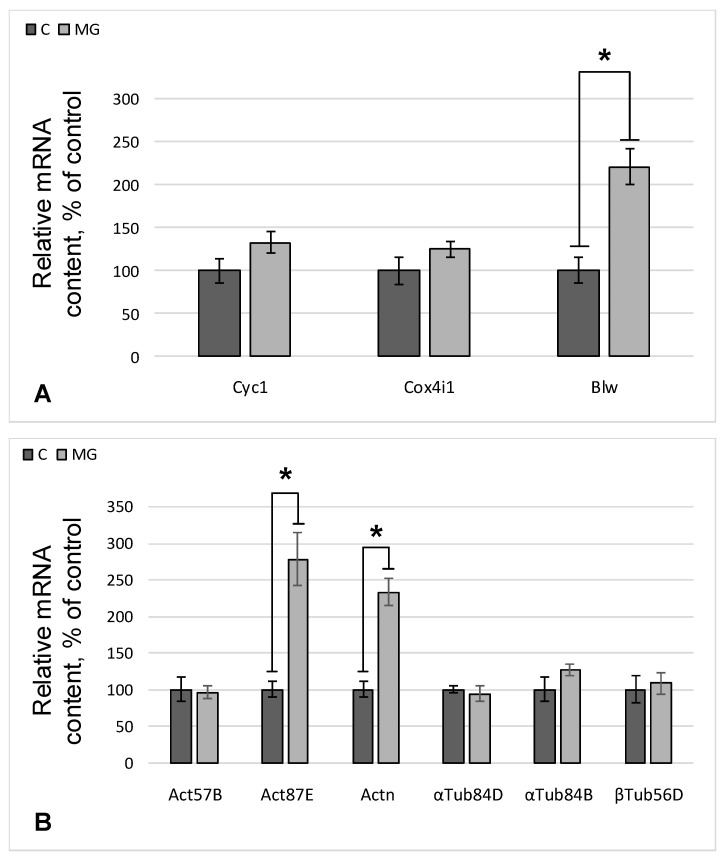
The relative content of mRNA. (**A**) mRNA of genes encoding proteins involved in the cellular respiration of mitochondria. Cyc1—mRNA of the gene encoding cytochrome c; Cox4i1—mRNA of the gene encoding cytochrome c oxidase; Blw—mRNA of the gene encoding the subunit of ATP synthase F1. (**B**) mRNA of genes that form the structures of the actin and tubulin cytoskeleton. Act57B and Act87E—mRNA of genes encoding beta-actin; Actn—mRNA of the gene encoding alpha-actinin; αTub84D and αTub84B—mRNA of genes encoding alpha-tubulin; βTub56D—mRNA of the gene encoding beta-tubulin; C—control group; MG—group of simulated microgravity. * *p* < 0.05 compared to control group C.

The relative content of Cyc1 and Cox4i1 mRNA did not change compared to the control, while the expression of the gene encoding the Blw protein increased by 121% (*p* < 0.05) in the simulated microgravity group (Figure 3A). The expression of genes encoding beta-actin and the actin-binding protein alpha-actinin increased by 178% (*p* < 0.05) and 133% (*p* < 0.05), respectively, after simulated microgravity conditions (Figure 3B). No changes in the expression of genes encoding alpha- and beta-tubulin were found in any of the groups (Figure 3B).

### 2.4. Histone Acetylation Relative Content

**Figure 4:**
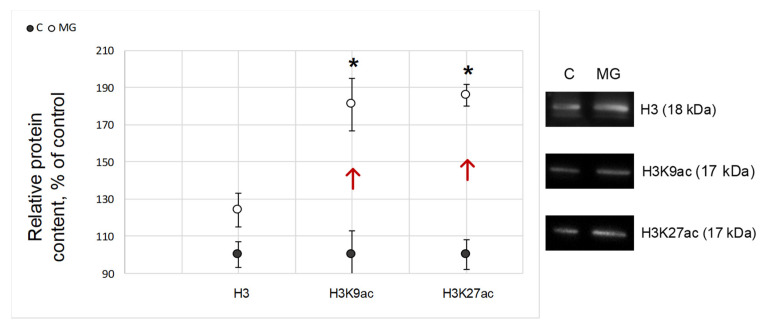
Relative content of proteins that form the nucleosomal core and participate in DNA organization in *Drosophila melanogaster* oocytes. H3 (18 kDa)—histone H3; H3K9ac (17 kDa)—histone H3 acetylated at Lys9; H3K27ac (17 kDa)—histone H3 acetylated at Lys27; C—control group; MG—simulated microgravity group. Arrows indicate the direction of change. * *p* < 0.05 compared with the control group C. Typical Western blots are shown on the right.

The relative content of histone H3 forming the nucleosomal core and involved in DNA organization did not change after exposure to simulated microgravity as compared to the control group (Figure 4). The levels of Lys9 (H3K9ac) and Lys27 (H3K27ac) acetylated histone H3 in the MG group were significantly higher compared to the control by 81% (*p* < 0.05) and 86% (*p* < 0.05), respectively (Figure 4).

## 3. Discussion

Studying the effect of microgravity on gametogenesis may be important for maintaining reproductive health during a long space flight. We analyzed cellular respiration as a parameter reflecting the functional state of the ovaries in the fruit fly *Drosophila melanogaster* under conditions of simulated microgravity during the full cycle of gametogenesis and estimated the content of proteins in the respiratory chain and various components of the cytoskeleton. The results obtained indicated that the intensity of cellular respiration of the ovaries in flies, the full development cycle of which occurred under simulated microgravity, was significantly higher than that in the controls. This may be due to an increase in the number of complexes of the mitochondrial respiratory chain, but we did not observe changes in the content of respiratory chain proteins, with the exception of the ATP synthase F1 (Blw) subunit (Figure 2A), the relative content of which increased; however, respiratory integrity is important for normal functioning complexes. Therefore, it can be assumed that the increase in oxygen consumption by the ovaries after maturation under simulated microgravity conditions is associated with a change in the intensity of electron transfer due to some complex of the respiratory chain.

Inhibitory analysis showed that such an increase is most likely due to complex I of the respiratory chain, as the rates of cellular respiration after inhibition of NADH dehydrogenase by rotenone and antimycin A did not differ from the control level (Figure 1).

The first complex of the electron transport chain can be activated by mitochondrial STAT3 [27] due to an increase in the actin-binding protein alpha-actinin in the cytoplasm [28]. Therefore, we determined the content of alpha-actinin and recorded an increase in its content. Moreover, in our previous study, after a short-term experiment under the simulated microgravity of *Drosophila melanogaster*, cellular respiration also increased, and we noted an increase in the content of alpha-actinin [29]. In addition, the relative content of beta-actin also increased. The actin cytoskeleton is involved in maintaining the localization of mitochondria, and the microtubule in their transport. During normal oogenesis, accumulation of mitochondria in the posterior part of the oocyte during oogenesis in *Drosophila melanogaster* begins at stage 10, increases up to stage 13, and persists during embryogenesis [30,31]. At the same time, we did not find any changes in the relative content of the main components of microtubules, alpha- and beta-tubulin (Figure 2B).

To examine the reasons for the increase in the content of actin and alpha-actinin, the relative content of mRNA of genes encoding cytoskeletal proteins was determined. As expected, the mRNA content of alpha- and beta-tubulin did not change, while for beta-actin and alpha-actinin, it increased (Figure 3B). Accordingly, a question arises concerning the reasons for this transcription increase.

Since DNA methylation in *Drosophila* is described but is not widely researched [32], and the RNA interference mechanism predominantly leads to the elimination of foreign/aberrant RNA [33,34], we decided to analyze the posttranslational modifications of histones, primarily the acetylation of histone H3, as one of the main participants in the regulation of gene expression due to its position in the nucleosoma.

The results indicate that in the ovaries of *Drosophila melanogaster*, the relative content of histone H3 acetylated at Lys9 and Lys27 significantly (*p* < 0.05) increased (Figure 4). Acetylation of histones by lysine is considered predominantly as a transcription-activating factor, since this posttranslational modification probably causes a decrease in the positive charge on the histone surface and increases the availability of DNA for transcription enzymes [35,36]. For plants, it was shown that an increase in the content of H3K9ac strongly correlates with an increase in actin expression [37]. For animals, the relationship appears to be more complex. Activation of the actin/MKL1 signaling pathway leads to an increase in the content of H3K9ac and throughout the genome [38]. In addition, the interaction of H3K27ac with the MAPK signaling pathway is a well-documented fact [39]. Therefore, it cannot be ruled out that the changes in expression observed by us may in some way be associated with a change in the acetylation status of histone H3 lysine residues, but this assumption requires further research. 

## 4. Materials and Methods

### 4.1. Study Design

Males and females of the Canton-S *Drosophila melanogaster* line at 2 days of age were placed in 50 mL Falcon tubes (30 individuals per tube) and confined using an air-permeable lid. To maintain the *Drosophila melanogaster*, a nutrient medium (water with the addition of 0.7% agar, 4% sugar, 4% semolina, 2.5% baker’s yeast and 1% propionic acid) at a volume of 15 mL was used. To simulate microgravity, a microgravity simulator (Gravite^®^, GC-US-RCE010001, Space Bio-Laboratories Co., Ltd., Hiroshima, Japan) was used, which provides a superposition of cell orientation in the gravity field equal to zero in an average of 15 s.

Two study groups were created:

− MG (simulated microgravity group), which was placed in conditions of simulated microgravity;
− C (control group), which was placed next to the simulator platform to ensure identical containment conditions.

One day after the start of the experiment, the air-permeable covers were removed and replaced after the emergence of mature individuals from the test tubes. Cultivation of test tubes continued throughout the full cycle of gametogenesis, which corresponded to 15 days under these conditions, until females reached 2 days of age. At the end of exposure, the flies of the experimental and control groups were subjected to ovarian extirpation.

All experimental procedures were approved by the Commission on Biomedical Ethics of the State Research Center of the Russian Federation-IBMP RAS (Protocol No. 521, dated 25 September 2019).

### 4.2. Measuring Cellular Respiration Using the Polarography Method

Twenty to thirty *Drosophila melanogaster* ovaries from each experimental group were used to analyze cellular respiration according to the protocol detailed by Kuznetsov et al. [40]. After isolation, the ovaries were incubated in a solution of saponin at a concentration of 10 μg/mL for 10 min at +25 °C in a shaker (Thermo Shaker PST-60HL-4, Biosan, Riga, Latvia). Next, the samples were transferred to a polarographic cuvette, and changes in the oxygen concentration were recorded using an Oxygraph+ polarograph (Hansatech Instruments, Ltd., Norfolk, UK) at +25 °C. The substrate inhibitor assay was performed according to the protocol of Kuznetsov et al. [40] with modifications detailed in our previous study [41].

When transferring the ovaries into a polarographic cuvette, the basal oxygen uptake rate, V_0_, was recorded. Then, substrates of NADH dehydrogenase, 10 mM glutamate and 5 mM malate were added, and the respiration rate of the first complex, V_glu+mal_, was recorded. After the addition of 2 mM ADP, the maximum respiration rate, V_max_, was recorded. With the sequential addition of 0.5 μM rotenone, an inhibitor of complex I, followed by 10 mM succinate, the substrate of succinate dehydrogenase, the rate of cellular respiration of the second complex, V_II_, was recorded. Next, a cytochrome c reductase inhibitor, 5 μM antimycin A, was added, followed by artificial substrates of cytochrome c oxidase, 0.5 mM TMPD + 2 mM ascorbate, after which the rate of cellular respiration of the fourth complex of the V_IV_ respiratory chain was recorded. After adding 10 μM cytochrome c to each sample, a test was performed for the integrity of the outer mitochondrial membrane: if the membrane remained intact, the rate of cellular respiration did not change relative to the V_IV_ rate or increased by a maximum of 15%. Only samples with an intact membrane were considered. The rate of cellular respiration was measured in pmol O_2_·mL^−1^·min^−1^ per ovary. For each experimental point, at least three biological replicates were tested.

### 4.3. Estimation of the Relative Protein Content by Western Blotting

To isolate proteins, frozen ovaries of each group were used, which were homogenized in a Laemmli buffer with a cocktail of protease inhibitors (Calbiochem, San Diego, CA, USA). Denaturing electrophoresis was performed on polyacrylamide gels using the Laemmli method (Bio-Rad Laboratories, Hercules, CA, USA). Based on the measured concentration (using NanoDrop One by Thermo Fisher Scientific, Waltham, MA, USA), an equal amount of protein was placed into each well, separated by electrophoresis, and transferred to a nitrocellulose membrane [42]. Efficiency of the protein transfer was controlled by Ponceau S staining before the milk-blocking membrane. To quantify each protein, specific primary monoclonal antibodies were used as shown in Table 1 at dilutions recommended by the manufacturer.

Horse antibodies conjugated with horseradish peroxidase for chemiluminescence detection (Cell Signaling Technology, Danvers, MA, USA # 7076S) at a dilution of 1:2000 were used as secondary antibodies for the detection of mouse IgG and for the detection of rabbit IgG-goat antibodies conjugated with horseradish peroxidase (Cell Signaling Technology, Danvers, MA, USA # 7074S) at a dilution of 1:2000. Next, the membranes were treated with SuperSignal ™ West Femto Maximum Sensitivity Substrate (Thermo Scientific, Waltham, MA, USA) and detected. Secondary goat antibodies (Sigma, St. Louis, MI, USA, # B7139) were used against rat IgG, and then the membrane was treated with a streptavidin solution conjugated with horseradish peroxidase (Sigma, St. Louis, MI, USA, # E2886) at a dilution of 1:10,000, after which the protein bands were identified with 3,3′-diaminobenzidine (Amresco, Solon, OH, USA, # E733-50).

The resulting protein bands were analyzed using the Fiji package (https://imagej.net/Fiji, accessed on 26 July 2021).

### 4.4. Estimation of the Relative Content of mRNA by Polymerase Chain Reaction (PCR)

Total mRNA was isolated from *Drosophila melanogaster* ovaries using the RNeasy Micro Kit (Qiagen, Germany) according to the instructions. Reverse transcription was performed using d (T) 15 as the primer, and 500 ng RNA was controlled by measuring the concentration. After reverse transcription, the amount of cDNA was measured in order to control the application of the same amount for qPCR. Real-time PCR was performed using primers selected via the Primer3Plus and Primer-BLAST (https://www.ncbi.nlm.nih.gov/tools/primer-blast/, accessed on 16 September 2020) (Table 2) to assess the expression levels of the studied genes. The specificity of primers was controlled by product size, melting curve, and negative controls without DNA. The 2(-Delta DeltaC(T)) method [43] with Gapdh normalization was used to determine the fold change. 

### 4.5. Statistical Analysis

For statistical analysis of the results, ANOVA and post hoc *t*-tests were used to assess the significance of differences between groups at a level of *p* < 0.05.

## 5. Conclusions

Summarizing the above, in the ovaries of *Drosophila melanogaster*, the full development cycle of which took place under simulated microgravity conditions, an increase in the acetylation of Lys9 and Lys27 in histone H3 occurred, which correlated with an increase in the expression of actin and alpha-actinin. Against the background of an increase in the relative content of the components of the actin cytoskeleton, an increase in the rate of cellular respiration due to complex I of the respiratory chain was observed.

## Figures and Tables

**Table 1:** Primary antibodies.

Primary Antibodies	Molecular Weight	Dilution	Producer	Catalog Number
Cytochrome *c*-1	13.5 kDa	5 μg/mL	Abcam, UK, Cambridge	#ab13575
Cytochrome *c* oxidase	16 kDa	1 μg/mL	Abcam, UK, Cambridge	#ab14744
ATP synthase F1 (Blw)	56 kDa	1 μg/mL	Abcam, UK, Cambridge	#ab14748
Alpha-tubulin	50 kDa	1:1000	Abcam, UK, Cambridge	#ab52866
Beta-tubulin	50 kDa	1:1000	Abcam, UK, Cambridge	#ab179513
Beta-actin	42 kDa	1:5000	Abcam, UK, Cambridge	#ab227387
Alpha-actinin	102 kDa	1 μg/mL	Abcam, UK, Cambridge	#ab50599
Histone H3	11 kDa	1:1000	Abcam, UK, Cambridge	#ab10799
Histone H3 Lys 9 acetylated	17 kDa	1:1000	Abcam, UK, Cambridge	#ab4441
Histone H3 Lys 27 acetylated	17 kDa	1 μg/mL	Abcam, UK, Cambridge	#ab4729

**Table 2:** Primer sequence and product size.

Gene	Primer Sequence, Forward/Reverse (5′…3′)	Product Size, bp
CG4769	GCAGCGACATTGCGAAGATT/ACTGCTCCAGGGCGTAGATA	181
CG10396	ACTGCCGTCGAAATGAGCTT/TCACGTAGGGCACACAACTC	227
Blw	AATAGGAGTAGCGGTGCGTG/AACCACGGATTGAAGGCGAT	201
Act57B	GCCTAGCACCAACACTAGCA/CGCGAGCGATTAACAAGTGG	288
Act87E	CCGAATACCGAAAGCCCACT/CTGGGCCTCATCACCAACAT	269
Actn	ACAAGCCGAACATTGAGGAG/GCGTTTCCATCGTGTAGTTG	96
alphaTub84D	AAGGACTACGAGGAGGTCGG/ATGCGAGTGGGAGCGTATGA	124
alphaTub84B	CACTGGTACGTTGGTGAGGG/CCCATCGAGCGTTGAAGTGG	166
betaTub56D	AAGCGGACAGTTTGTGTTGTG/ACCAGCTTGGATGTGAACGA	115
Gapdh	ATACTCATCAACCCTCCCCC/GGCTGAGTTCCTGCTGTCTT	142

## Data Availability

All data generated or analyzed during this study are included in this article.
